# Relationship Between Adherence to Antihypertensive Medication Regimen and Out-of-Pocket Costs Among People Aged 35 to 64 With Employer-Sponsored Health Insurance

**DOI:** 10.5888/pcd16.180381

**Published:** 2019-03-21

**Authors:** Madeleine M. Baker-Goering, Kakoli Roy, David H. Howard

**Affiliations:** 1 Office of the Associate Director for Policy, Centers for Disease Control and Prevention, Atlanta, Georgia; 2 Department of Health Policy and Management, Rollins School of Public Health, Emory University, Atlanta, Georgia

## Abstract

We used administrative claims data from 2014 on people with employer-sponsored health insurance to assess the proportion of patients taking antihypertensive medications, rates of nonadherence to these medication regimens, and out-of-pocket costs paid by patients. We performed multivariate logistic regression analysis to examine the association between out-of-pocket costs and nonadherence. Results indicated that patients filled the equivalent of 13 monthly prescriptions and paid $76 out of pocket over the calendar year; the likelihood of nonadherence increased as out-of-pocket costs increased (adjusted odds ratios ranged from 1.04 to 1.78; *P* < .001). These findings suggest a need for improvement in adherence among patients with employer-sponsored insurance.

**Summary**

**What is already known about this topic?**

Only 40% to 74% of patients treated for hypertension take medications as prescribed. Patients’ out-of-pocket costs for medications can affect medication adherence.

**What is added by this report?**

We found a substantial proportion of people aged 35 to 64 with employer-sponsored health insurance to be nonadherent to antihypertensive medications (41%). People paid an average $6 for a 30-day supply of medication in 2014, and those paying higher out-of-pocket costs had a greater likelihood of being nonadherent.

**What are the implications for public health practice?**

The results highlight room for improvement in medication adherence among patients with employer-sponsored health insurance, even if facing small out of pocket costs.

## Objective

Hypertension is a leading risk factor for cardiovascular disease, and only 40% to 74% of people with diagnosed hypertension are adherent to prescribed medication (1–3). Nonadherence can result in uncontrolled hypertension, which increases the risk of acute cardiovascular disease events (4,5). People who take many different drugs, experience side effects from hypertension medication, have comorbidities, or face high out-of-pocket costs are more likely to be nonadherent (6). No recent studies among the privately insured population of the United States describe rates of nonadherence and actual out-of-pocket payments for antihypertensive medications.

## Methods

We used 2014 administrative claims data from the IBM MarketScan Commercial Database (IBM Corp), which provided de-identified health care claims data for enrollees and their dependents in employer-sponsored health insurance plans. We limited the study sample to adults aged 35 to 64 who were continuously enrolled with prescription drug coverage in 2014 (N = 3,362,633). We focused on 2014 after using the IBM Treatment Pathways online tool (IBM Corp), which reported stable trends of proportions of patients with any hypertension diagnosis (25%−26%) and proportions of patients filling an antihypertensive medication prescription (29%−30%) in 2010 through 2014.

We identified people with hypertension by the appearance of *International Classification of Diseases, 9th Revision* (ICD-9), diagnosis codes (401.x–405.x) on at least 2 outpatient or 1 inpatient or emergency department claim, and we identified cardiovascular disease events resulting in an inpatient or emergency department visit by using ICD-9 codes from the Center for Medicare and Medicaid Services’ Chronic Conditions Warehouse for ischemic heart disease, acute myocardial infarction, cerebrovascular disease, and heart failure (398.91, 402.x–404.x, 410.x, 411.x–413.x, 414.0x, 414.12, 414.2, 414.3, 414.8, 414.9, 428.x, 430.x, 431.x, 433.x1, 434.x, 436.x, 997.02) (7). We measured adherence to antihypertensive medications among enrollees with at least 1 antihypertensive drug claim in 2014. We identified antihypertensive medications by Redbook drug codes for therapeutic class (except for angiotensin II receptor blockers, which we identified by using National Drug Codes, because they were not identified in Redbook by therapeutic class) (8). We measured the proportion of days covered (days covered equaled the patient’s supply of medication from the day of the first filled prescription through the end of the calendar year), divided by the number of days in that same period, calculated for each medication class, and then averaged across number of medication classes per person. Enrollees with less than 80% of days covered were considered nonadherent, a cutoff used in many studies (4,9). We report total out-of-pocket cost (sum of copayments, coinsurance, or deductibles) and total payments for antihypertensive medications. We also reported number of filled prescriptions and out-of-pocket and total medication costs for a 30-day equivalent supply of antihypertensive medications (eg, 15 days of supply becomes 0.5 of a 30-day fill). Out-of-pocket costs was converted to a categorical variable ($0, <$5, $5–<$10, $10–<$15, $15–<$20, $20–<$50, and ≥$50).

We used multivariate logistic regression to estimate the association between category of out-of-pocket cost and the likelihood of being nonadherent and presented these data as odds ratios. We used Stata 12 SE (StataCorp, LLC) to analyze data.

## Results

In 2014, 22% of adults aged 35 to 64 (N = 2,897,548) were diagnosed with hypertension (Table 1). Among the 27% (N = 3,462,582) of adults who filled prescriptions for antihypertensive medications, 41% (N = 1,428,298) of those who filled a prescription were nonadherent to their antihypertensive medication regimen. Nonadherence decreased with age and was higher for women than men and for people using branded medications than those using generic ones. Regionally, nonadherence was highest among people living in the South.

**Table 1:** Characteristics of Sample of Adults Aged 35 to 64 (N = 3,362,633), Study of Relationship Between Nonadherence to Antihypertensive Medication (AHM) Regimen and Enrollment in an Employer-Sponsored Health Insurance Plan, United States, 2014^a^

Variable	No. Enrollees	Diagnosed With Hypertension, N (%)	Treated With AHM, N (%)	Nonadherent Among Treated, N (%)	Number of AHM Prescriptions Filled^b^^,^ c	Out of Pocket Cost, AHM, $^d^		Total Cost, AHM, $^b^^,^ e	
						30-Day Supply	Annual Supply	30- Day Supply	Annual Supply
**Total **	13,035,703	2,897,548 (22)	3,462,582 (27)	1,428,298 (41)	13.2	5.78	76.24	17.34	228.57
**Age, y**									
35–44	4,067,167	436,240 (11)	576964 (14)	3,187,186 (52)	10.1	5.93	59.67	17.02	171.15
45–54	4,759,074	10,21,153 (21)	1,237,594 (26)	2,756,655 (43)	12.5	5.86	73.22	17.36	217.02
55–64	4,209,462	1,440,155 (34)	1,648,024 (39)	2,409,621 (36)	14.8	5.70	84.30	17.40	257.35
**Sex**									
Male	6,073,363	1,469,555 (24)	1,690,278 (28)	2,442,685 (40)	13.7	5.81	79.55	17.67	241.87
Female	6,962,340	1,427,993 (21)	1,772,304 (25)	2,448,195 (43)	12.7	5.75	73.08	16.99	215.89
**Insurance plan^f^**									
PPO	7,438,985	1,806,612 (24)	1,996,135 (27)	2,286,115 (41)	13.2	5.84	77.14	18.26	241.06
HMO	1,342,925	290,178 (22)	351,450 (26)	3,261,165 (43)	13.1	6.23	81.77	16.45	216.07
CD/HD	2,158,440	445,098 (21)	519,476 (24)	3,159,601 (42)	12.8	5.42	69.40	15.35	196.63
Other^g^	1,240,828	333465 (27)	372,064 (30)	3,243,771 (41)	13.5	5.94	80.44	16.54	223.83
**Region^h^**									
Northeast	2,708,175	503,112 (19)	665,342 (25)	3,047,219 (38)	13.7	5.13	70.18	17.02	232.63
Midwest	2,685,343	577697 (22)	708,806 (26)	3,025,079 (38)	13.6	5.14	70.04	14.57	198.38
South	5,156,805	1,378,328 (27)	1,533,458 (30)	2,604,672 (44)	12.9	6.40	82.32	19.13	246.10
West	2,156,128	345,237 (16)	454,118 (21)	3,196,654 (41)	12.9	5.23	67.36	15.68	202.14
**Geographic area**									
Urban	11,269,871	2,441,266 (22)	2,810,705 (25)	1,807,038 (41)	13.2	5.66	74.41	17.33	228.05
Rural	1,765,832	456,282 (26)	551,928 (31)	3,141,037 (42)	13.3	6.00	79.94	16.87	224.55
**Type of AHM used^i^**									
Generics only	3,041,215	1,855,141 (61)	3,041,215 (100)	1,216,486 (40)	12.8	4.41	56.45	10.72	137.23
Ever use branded	421,367	290,743 (69)	421,367 (100)	223,325 (53)	16.1	13.60	219.03	40.73	655.75

Abbreviations: CD/HD, consumer driven or high deductible health plan; HMO, health maintenance organization; HTN, hypertension; PPO, preferred provider organization.

a Data are from IBM’s MarketScan Commercial Database.

b Outliers (negative values and ≥99th percentile for annual AHM cost and total payment) and missing values excluded.

c 30-day equivalent fills.

d This includes copayments, coinsurance, and deductibles.

e Total payments include all payments made, including insurer payments, copayments, coinsurance, deductibles, and coordination of benefits payments.

f Plan type was missing for 7% of the sample.

g Other includes comprehensive, exclusive provider organization, and point of sale plans.

h Region was missing for 3% of the sample.

i This splits AHM users into 2 mutually exclusive groups: those who only filled prescriptions for generic AHMs in 2014 (88%) and those who ever filled a prescription for a branded AHM in 2014 (12%).

Patients filled an average of 13 monthly antihypertensive medication prescriptions during the calendar year and paid on average $5.78 out of pocket per 30-day supply and $76 annually. Total costs (patient and insurer) for antihypertensive medications were $17 per 30-day supply and $229 annually. People who used branded medications had the highest out-of-pocket costs ($13.60 per 30-day supply) and highest total costs ($40.73 per 30-day supply). Patients in health maintenance organizations had higher out-of-pocket costs that those in other types of insurance plans. Residents of rural areas also had higher out-of-pocket costs than those paid by those in urban areas, but they used less expensive medications.

About 90% of patients incurred out-of-pocket costs for medications, but 83% paid less than $10 per 30-day supply of antihypertensive medications. However, 30% of patients paid the full costs of their medications (approximately $41 in annual out-of-pocket costs for an average of 10 fills during the calendar year.).

We calculated the unadjusted and multivariate-adjusted odds ratios for the association between nonadherence and patient characteristics (Table 2). The likelihood of nonadherence increased as out-of-pocket costs increased (odds ratios, compared with those with no out-of-pocket costs, ranged from 1.04 for those paying less than $5, to 1.78 for those paying more than $50).

**Table 2:** Odds of Nonadherence to an Antihypertensive Medication Regimen in Relation to Out-of-Pocket Costs, Adults Aged 35 to 64 Enrolled in an Employer-Sponsored Health Insurance Plan, United States, 2014^a^

Variables	Unadjusted Odds Ratio (95% CI)	*P* Value	Adjusted Odds Ratio^b ^(95% CI)	*P* Value
**Out-of-pocket cost, 30-day supply of antihypertensive medication, $**				
0	1 [Reference]			
<5	0.99 (0.98–1.00)	.003	1.04 (1.04–1.05)	<.001
5–<10	1.35 (1.34–1.37)	<.001	1.36 (1.35–1.37)	<.001
10–<15	1.60 (1.58–1.61)	<.001	1.51 (1.49–1.52)	<.001
15–<20	1.77 (1.74–1.79)	<.001	1.50 (1.48–1.52)	<.001
20–<50	1.89 (1.87–1.92)	<.001	1.44 (1.42–1.46)	<.001
≥50	2.45 (2.39–2.52)	<.001	1.78 (1.73–1.83)	<.001
**Age, y**				
35–44	1 [Reference]			
45–54	0.69 (0.68–0.69)	<.001	0.69 (0.68–0.69)	<.001
55–64	0.52 (0.51–0.52)	<.001	0.51 (0.51–0.51)	<.001
**Sex**				
Male	1 [Reference]			
Female	1.14 (1.13–1.14)	<.001	1.14 (1.14–1.15)	<.001
**Hypertension diagnosis in 2014**				
No	1 [Reference]			
Yes	0.89 (0.89–0.90)	<.001	0.88 (0.87–0.88)	<.001
**Cardiovascular disease event in 2014**				
No	1 [Reference]			
Yes	2.00 (1.97–2.02)	<.001	2.12 (2.09–2.15)	<.001
**Type of AHM used^c^**				
Generic AHMs only	1 [Reference]			
Any use of branded AHMs	1.72 (1.71–1.73)	<.001	1.52 (1.51–1.52)	<.001
**Type of insurance plan**				
PPO	1 [Reference]			
HMO	1.07 (1.06–1.08)	<.001	1.07 (1.06–1.08)	<.001
CD/HD	1.03 (1.02–1.03)	<.001	1.05 (1.04–1.06)	<.001
Other	1.01 (1.00–1.01)	.15	1.04 (1.03–1.04)	<.001
**Region**				
Northeast	1 [Reference]			
Midwest	1.03 (1.02–1.04)	<.001	1.02 (1.02–1.03)	<.001
South	1.31 (1.30–1.31)	<.001	1.24 (1.23–1.25)	<.001
West	1.18 (1.17–1.19)	<.001	1.16 (1.15–1.17)	<.001
**Geographic region**				
Rural	1 [Reference]			
Urban	0.98 (0.97–0.98)	<.001	0.99 (0.98–1.00)	.002

Abbreviations: CD/HD, consumer driven or high deductible health plan; CI, confidence interval; HMO, health maintenance organization; PPO, preferred provider organization.

a Data are from IBM’s MarketScan Commercial Database.

b Adjusted for variables listed in the table.

c This splits AHM users into 2 mutually exclusive groups: those who only filled prescriptions for generic AHMs in 2014 (88%) and those who ever filled a prescription for a branded AHM in 2014 (12%).

The likelihood of nonadherence was greatest among patients who used branded antihypertensive medications and those living in the South and was smallest among those with a hypertension diagnosis in 2014 and patients aged 55 to 64. When we restricted the sample to patients with a hypertension diagnosis as a sensitivity analysis, the associations were similar in magnitude and significance.

## Discussion

We found that nonadherence to antihypertensive medication regimens was common and was most common among patients with higher out-of-pocket costs. A 2016 study estimated that patients with commercial insurance paid about $4.13 in copayments per antihypertensive medication prescription filled in 2014, slightly lower than the out-of-pocket cost estimate reported in our study, which provides a more comprehensive estimate that includes copayments, coinsurance, and deductibles (10).

Numerous experimental and quasi-experimental studies have found a causal relationship between lowering patients’ out-of-pocket costs and reducing medication nonadherence (11). Our study shows an association between out-of-pocket costs and nonadherence among enrollees in employer-sponsored insurance plans. However, nonadherence is influenced by many other factors unrelated to cost, such as number of pills to be taken (eg, 1 daily medication versus combination medications) or the burden of filling prescriptions (eg, increasing the number of doses per prescription, delivering prescriptions by mail) (6,10,12). The data we used were collected for administrative purposes and were not nationally representative. In addition, claims data have many documented limitations, including that prescriptions filled do not measure actual medication used.

Our study findings show that there is room for improving adherence to antihypertensive medications among patients with employer-sponsored insurance and that patients who faced higher out-of-pocket costs had a greater likelihood of being nonadherent.
